# A comparative analysis and critical review of two rTMS treatment durations in dogs with drug-resistant idiopathic epilepsy

**DOI:** 10.1186/s12917-026-05295-0

**Published:** 2026-01-19

**Authors:** Delia Hünting, Sebastian Meller, Friederike Twele, Nina Meyerhoff, Sofie F.M. Bhatti, Holger A. Volk, Marios Charalambous

**Affiliations:** 1 Department of Small Animal Medicine & Surgery, University of Veterinary Medicine Hannover, Hannover, Germany; 2 Center for Systems Neuroscience Hannover, Hannover, Germany; 3 Department of Small Animals, Faculty of Veterinary Medicine, Ghent University, Ghent, Belgium

**Keywords:** Dogs, Neurostimulation, Epilepsy, Seizures, Refractory

## Abstract

**Background:**

Repetitive transcranial magnetic stimulation (rTMS) is an effective, non-invasive management option for canine drug-resistant idiopathic epilepsy, but optimal stimulation protocols have not yet been established.

**Hypothesis/objectives:**

The hypothesis is that treatment duration influences the therapeutic outcome of rTMS in dogs with drug-resistant idiopathic epilepsy. The objective is to compare and critically review the effect of different rTMS treatment durations on clinical outcomes in these patients.

**Methods:**

Two previously published rTMS protocols (5-day vs. 3-day), using the same total number of pulses and a coil output of ≥ 70% were reviewed. The primary difference between the stimulation protocols did lay in the daily number of pulses administered. In the five-day protocol, 1620 pulses were delivered daily with a stimulation duration of approximately one hour, whereas the three-day protocol involved administering 2700 pulses per day, with the stimulation lasting around 1 h and 40 min. The monthly seizure frequency (MSF) and monthly seizure day frequency (i.e. days with seizures, MSDF) of the two study groups (5-day protocol, *n* = 12; 3-day protocol, *n* = 15) were compared in terms of the magnitude of change in seizure activity over a three-month period before and after rTMS stimulation.

**Results:**

Both stimulation protocols were considered effective and resulted in an overall reduction in MSF. However, the 5-day protocol showed a significantly more pronounced reduction in MSF, with a mean decrease of 44%, compared to 19% in the 3-day protocol. The 3-day protocol had a lower responder rate than seen in the 5-day rTMS-group. Neither stimulation protocol did aggravate the progression of epilepsy.

**Conclusion:**

rTMS is a safe treatment method, with no persistent adverse effects observed in any of the animals. The number of repetitions of the therapeutic intervention might influence the rTMS efficacy, which should be considered when establishing new rTMS protocols.

## Background

Idiopathic epilepsy (IE) is the most common neurological disease in humans and dogs, characterised by recurrent spontaneous epileptic seizures [1–3]. The lifelong nature of the disease significantly impairs the quality of life for both affected dogs and their owners due to epileptic seizures and adverse effects associated with antiseizure medications (ASMs) [4]. Consequently, affected animals are more likely to be euthanised, resulting in a significantly reduced lifespan [5]. Many patients do not respond adequately to ASMs and are therefore classified as drug-resistant. Drug-resistant epilepsy (DRE) is defined as the inability to control seizure activity in an individual despite the administration of two or more ASMs within the therapeutic range or at maximally tolerated doses [6, 7]. To date, therapeutic plasma concentrations have been established only for phenobarbital and potassium bromide in dogs [8], and dosage alone provides limited information due to considerable interindividual variability in plasma levels. Additional ASMs might not improve seizure control [9]. A recent panel of human medical and veterinary neurologists and neuropharmacologists, however, suggested that there is still a realistic, but smaller chance of achieving adequate seizure control if additional drugs are added [10], representing the current practice in human medicine.

In canine epilepsy, several factors may contribute to drug resistance and variability in therapeutic response including but not limited to (i) genetic variants that could affect drug transport or neuronal excitability, (ii) neuroinflammatory processes that could modulate blood-brain barrier function or drug transporter expression, and (iii) metabolic disturbances or were misdiagnosed epilepsy [6, 9]. A recent study revealed that approximately one-third of patients initially diagnosed with DRE had other underlying causes for their drug resistance or were misdiagnosed with epilepsy, characterizing [11]; the majority of these dogs might respond positively to adjustments in the initial therapy protocol [11]. Therefore, pseudoresistance should also be considered and excluded before the diagnosis of DRE is established.

Drug-resistant epilepsy has been associated with certain breeds, such as the Australian Shepherd, Border Collie, Greater Swiss Mountain Dog, Cane Corso, as well as with factors such as a younger age at disease onset, being male, the occurrence of cluster seizures, and status epilepticus [6, 12–15]. The limited therapeutic efficacy and potential adverse effects of ASMs highlight the need for alternative or adjunctive treatment strategies beyond the pharmacologically grounded state of the art.

Repetitive transcranial magnetic stimulation (rTMS) is a non-invasive neuromodulation technique that has been investigated primarily in human medicine, where it is considered a safe and well-tolerated method to reduce cortical excitability, particularly when applied at low frequencies (≤ 1 Hz) [16]. The underlying mechanism can be multifactorial and may involve the induction of electric currents in cortical tissue via rapidly changing magnetic fields, leading to long-term depression (LTD)-like effects on neuronal activity. In human patients with IE, various rTMS protocols have shown promising results, although outcomes are influenced by stimulation parameters such as frequency, intensity, coil type, and stimulation site [17]. In veterinary medicine, however, data remain limited, and controlled studies in dogs are scarce [18, 19]. Initial pilot studies suggest that rTMS may be feasible and safe in canine patients, but further research is needed to determine its efficacy and optimize stimulation protocols for use in dogs.

Repetitive TMS has been applied in two former studies to manage DRE in dogs successfully, introducing a five-day and a three-day stimulation protocol [18, 20]. The main difference between the protocols was the daily number of pulses: 1620 pulses for 1 h/d in the five-day protocol [18] versus 2700 pulses for 1 h and 40 min/d in the three-day protocol [20], yielding the same total number of pulses in both protocols (*n* = 8100). A three-month period before and after stimulation was used to collect seizure data for comparisons between active and sham group. Notably, both studies resulted in significant reductions in seizure activity three months before versus three months after rTMS, but it remained unclear which protocol was more effective. The current study aims on an exploratory approach to compare the historical data of the two aforementioned protocols and to evaluate the safety of rTMS.

## Materials and methods

Both studies were reviewed and approved by animal and welfare committees, in Ghent [18] by the University’s ethical committee (EC 2016/30), and in Hannover [20] by the governmental animal and welfare committees in Lower Saxony (LAVES; reference number, 33.8-42502-04-22-00114). Owners were required to provide written informed consent for their dogs to participate in the studies.

### Animals

Dogs with DRE, regardless of age, breed, or sex, were included in both studies. The definition, classification, and diagnostic criteria for IE were based on the guidelines established by the International Veterinary Epilepsy Task Force (IVETF) [21, 22]. Drug-resistance was specified as epilepsy characterised by a less than 50% decrease in monthly seizure frequency (MSF), despite treatment with a minimum of two ASMs at an optimal dose and serum drug concentrations [7]. All dogs received phenobarbital and potassium bromide, and some dogs were additionally treated with other anti-seizure medications.

### Data sources

The Ghent study [18] comprised both a single-blinded and a non-blinded component, whereas the recently published Hannover study [20] reported only the single-blinded component. The non-blinded part of the Hannover study represented a subsequent study phase that was analyzed separately and had not been published previously. In the present analysis, this previously unpublished non-blinded component was included, which accounts for the difference in the total number of animals compared to the Hannover study.

### rTMS procedure

The procedures in both studies were largely identical. Two single-blinded, placebo-controlled studies were conducted, one in Ghent [18] and one in Hannover [20], each with 12 and 17 dogs, respectively. Seizure data were collected over a three-month period before and after stimulation. Sedation was administered using dexmedetomidine, butorphanol, and midazolam. The magnetic round coil was placed over the vertex of the dogs’ cranium. The stimulation parameters were the same in both groups, with a low frequency of 1 Hz (1 pulse per second), 90 pulses per train, and an intertrain interval of 120 s (Table 1). However, the number of trains differed between the two studies (1620 pulses/d vs. 2700 pulses/d), affecting the duration of daily stimulation (1 h vs. 1 h 40 min). The total number of pulses administered remained the same in both groups (8100). Two dogs from Hannover were euthanised during the study period due to poorly controlled seizures, resulting in insufficient seizure data for these animals. Seizure data of generalised tonic-clonic seizures were recorded by the owners in diaries, and no changes were made to the ASMs except for the administration of emergency medications in the case of a cluster seizure or status epilepticus.

**Table 1:** Stimulation parameters

	5-day protocol [18]	3-day protocol [20]
Frequency	1 Hz	1 Hz
Trains	18	30
Pulses per train	90	90
Intertrain interval	120 s	120 s
Pulses per day	1620	2700
Pulses per protocol	8100	8100
Daily duration	1 h	1 h 40 min
Coil output	based on motor cortex threshold; median 70% (range 70–90%)	based on motor cortex threshold; median 85% (range 70–100%)

### Statistical analysis

Microsoft ^®^ Excel 2022 and GraphPad Prism 10.4.1 (GraphPad Software, Inc., La Jolla, CA, USA) were used for data preparation, visualisation, and statistical analysis. The change in MSF (monthly seizure frequency) and MSDF (monthly seizure day frequency) relative to the 3-months pre-rTMS period was calculated and the relative average decrease in seizures across all animals within each protocol was reported. The Mann-Whitney U test was used to compare the relative change in MSF between both protocols after rTMS. Canines were classified as responders with a seizure activity reduction of ≥ 50%. A chi-square test was performed to assess differences in responder rates between both protocols. Regression analyses of cumulative seizure frequency and 95% confidence intervals (95%CIs) along the project timelines in weeks were conducted to examine temporal seizure dynamics in more detail before and after rTMS, specifically in improving and non-improving dogs of the 3-day protocol. Analysis of covariance (ANCOVA) was used to compare slopes (rates of cumulative seizure increase per week). All tests were two sided and a *P*-value of < 0.05 was considered significant.

## Results

The signalment and characteristics of the dogs included in the study can be found in Table 2.

**Table 2:** Baseline characteristics and medication of dogs in the 5-day and 3-day protocol group

	5-day protocol [18]	3-day protocol [20]
Number of dogs	12	15
Breed	Golden Retriever (2), Cane Corso (2), Australian Shepherd, Border Collie, French Bulldog, Jack Russel Terrier, Italian Spinone, Beagle, Boston Terrier, American Staffordshire Terrier	Mixbreed (6), Australian Shepherd (2), Great Swiss Mountain Dog, Samoyed, Mini Australian Shepherd, St. Bernard, Giant Schnauzer, Longhaired Collie, Siberian Husky
Age (years)	Median 4.1 (range 1.9–9)	Median 4.5 (range 1.5–8.5)
Sex/neuter status	Male (intact) *n* = 7 (58%) Female (neutered) *n* = 5 (42%)	Male (intact) *n* = 6 (40%) Male (neutered) *n* = 5 (33%) Female (neutered) *n* = 4 (27%)
IVETF-classification [21]	Tier I = 3 dogs (25%) Tier II = 9 dogs (75%)	Tier I = 5 dogs (33%) Tier II = 10 dogs (67%)
Medication	PB/KBr = 8 dogs (67%) PB/KBr/Leve = 4 dogs (33%)	PB/KBr = 6 dogs (40%) PB/KBr/Leve = 4 dogs (27%) PB/KBr/Imp (7%) PB/KBr/Leve/Imp (7%) PB/KBr/Topiramate (7%) PB/KBr/Gabapentin (7%) PB/KBr/Imp/Zonisamide (7%)
Plasma drug concentrations (mg/l)	PB: Median 33.5 (range 25–42), (therapeutic range 15–35) KBr: Median 1551 (range 925–2584), (therapeutic range 1000–2000)	PB: Median 27.6 (range 22.7–33.9), (therapeutic range 15–35) KBr: Median 1418 (range 1017–2297), (therapeutic range 1000–2000)

**Fig. 1:**
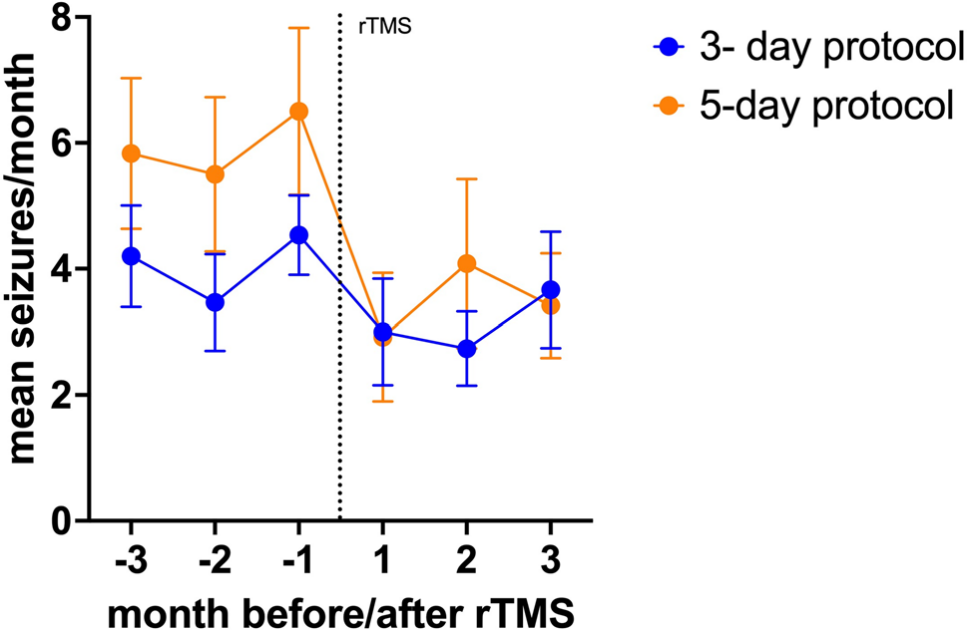
Orange and blue data points represent mean seizure frequencies and SD per month (y-axis) for the 5-day and 3-day protocols, respectively, with the x-axis representing the timeline in months before and after repetitive transcranial magnetic stimulation (rTMS) (dotted vertical line)

**Fig. 2:**
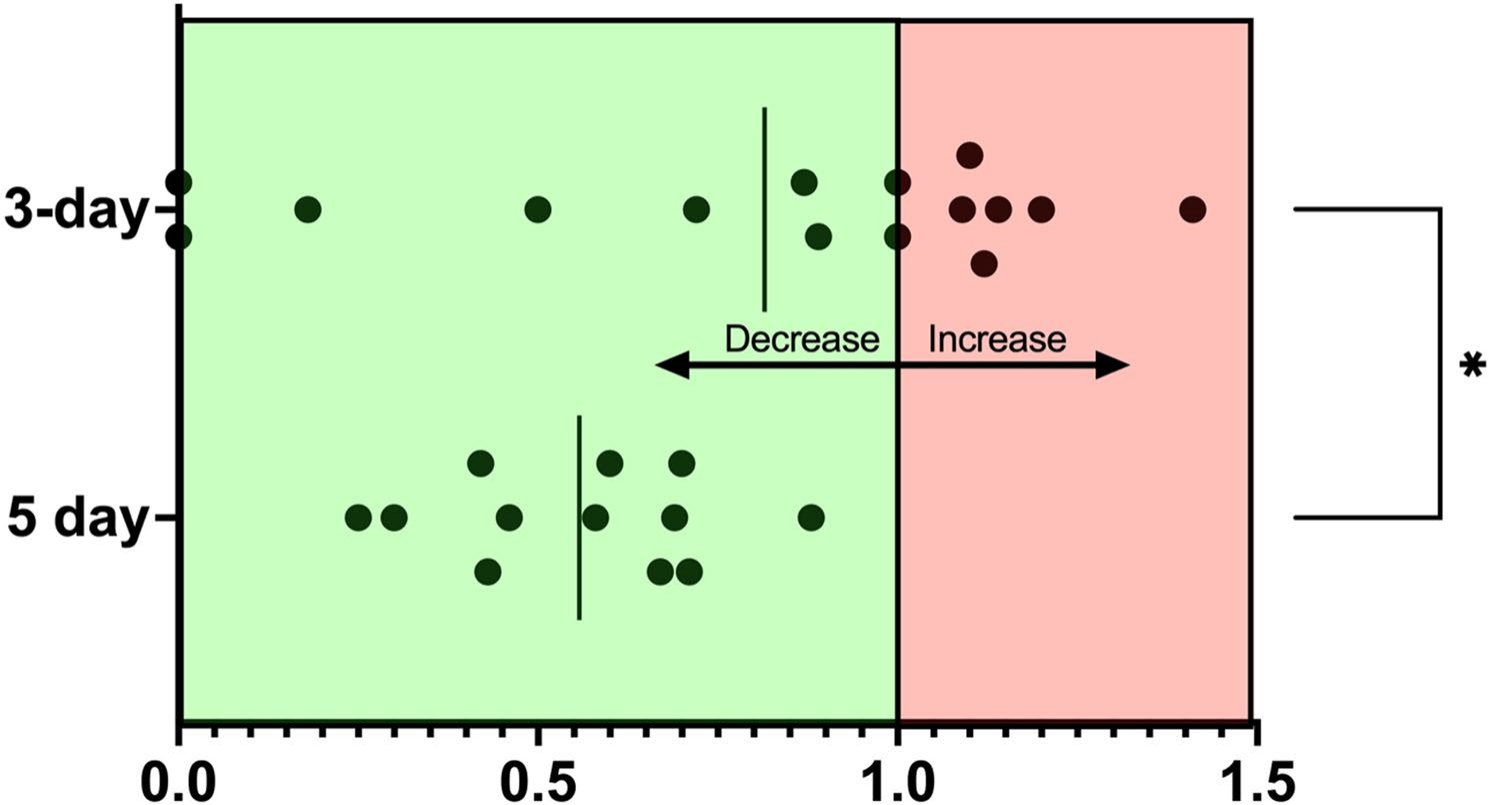
General increase/decrease of seizure frequency as ratio showing change in individual animals (represented as black data points) after the 5-day or 3-day repetitive transcranial magnetic stimulation (rTMS) protocol, with the respective pre rTMS seizure frequency as reference. A value of 1 indicates that the seizure frequency remained unchanged compared to the pre-stimulation period. A score of 0.5 indicates a 50% reduction in seizures, whereas a score of 0.0 indicates seizure freedom. Any value greater than 1.0 indicates an increase in seizure frequency compared to the pre-stimulation period. The vertical lines represent the mean of each group, respectively. All animals in the 5-day rTMS group showed a decrease in seizure frequency, but none became seizure-free. In contrast, the values in the 3-day group are more dispersed, with two animals showing seizure freedom, but also several animals showing an increase in seizure frequency. The decrease in seizure frequency was significantly more pronounced in the 5-day group compared to the 3-day group after rTMS (Mann-Whitney U test, *p = 0.02)

In the 5-day protocol group, animals were presented with a mean baseline seizure frequency of 5.7 (± 3.7 SD) seizures per month. In contrast, the group subjected to the 3-day protocol displayed a lower seizure frequency rate, averaging 4.0 (± 1.8 SD) seizures per month. However, this difference was not statistically significant (*p* = 0.11), indicating comparable baseline seizure frequencies between the groups. When comparing post-rTMS seizure frequency reduction within the two groups, it was observed that the 5-day regimen resulted in an average drop of 44% in MSF (MSDF − 42%) resulting in mean MSF of 3.5 (+/- 2.8 SD) (Fig. 1). On the other hand, the 3-day regimen demonstrated a more moderate decline of 19% in MSF (MSDF − 22%) with mean MSF of 3.1 (+/- 2.5 SD), which was significantly less than what was seen in the 5-day protocol (*p* = 0.02) (Figs. 1 and 2; Table 3).

However, none of the dogs in the 5-day protocol group achieved seizure freedom post-stimulation in contrast to the 3-day protocol (20%). In the 5-day protocol, 5 out of 12 dogs (42%) and 6 out of 12 dogs (50%) had an MSF or MSDF reduction of 50–100%, respectively, and could therefore be characterised as responders (Table 3). In the 3-day protocol, on the other hand, 7 out of 15 dogs (47%) showed a decrease in their MSF, with four dogs being classified as responders. An increase in seizure frequency was observed in 6 out of 15 dogs, while 2 dogs showed no change. The responder rate was significantly higher in the 5-day protocol, as determined by the chi-square test *(p* < 0.0001).

**Fig. 3:**
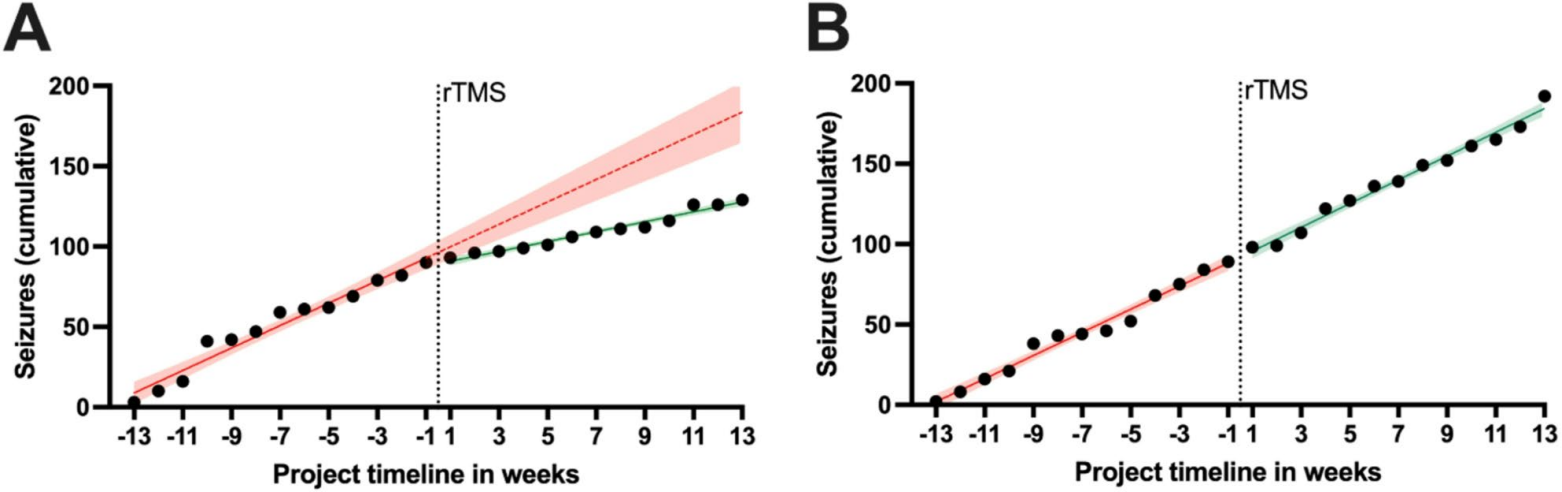
The overall cumulative seizure count of both dog groups having an improvement in seizure control (**A**) (*n* = 7) and dogs having a stagnation or deterioration in seizure control (**B**) (*n* = 8) were plotted for the 3-day group, with the repetitive transcranial magnetic stimulation (rTMS) being represented by the vertical lines (timepoint 0). (**A**) Dogs with improvement in seizure control showed a linear increase in seizure accumulation pre and post rTMS with excellent linear model fits (red solid line, R^2^ = 0.96 and green line, R^2^ = 0.97, respectively). During the pre rTMS phase, the increase was approximately 7.0 seizures/week (red solid line), and it declined to an increase of 3.1 seizures/week post rTMS (green line), which was significantly slower (Analysis of covariance, *p* < 0.0001). The red dotted line represents the extrapolation of the pre rTMS dynamics and highlights the divergence between both models. (**B**) Similarly, dogs not indicating an improvement showed excellent linear model fits with R^2^ = 0.98 for both the pre rTMS phase as well as the post rTMS phase. Their rates in seizure count increase were unchanged (+ 7.2 versus + 7.4 seizures/week, respectively). Shaded areas around the model lines and their extrapolation represent 95% confidence intervals

To further analyse the overall relative increase in seizure parameters in dogs not showing improvement from the 3-day protocol, linear regression analyses were conducted as part of a safety evaluation (Fig. 3). Overall, dogs with improvement in seizure frequency had a linear cumulative seizure increase of + 7.0 seizures/week before rTMS (95%CI 6.0–8.0; R^2^ = 0.96) compared to an increase of + 3.1 seizures/week after rTMS (95%CI 2.7–3.5; R^2^ = 0.97), yielding a significantly slower linear increase in seizure count per week after rTMS (ANCOVA, F(1, 22) = 68.18, *p* < 0.0001) (Fig. 3A). In contrast, dogs not improving showed an overall linear pre rTMS cumulative seizure increase of + 7.2 seizures/week (Confidence Intervals?; R^2^ = 0.98) versus an increase of + 7.4 seizures/week post rTMS (Confidence Intervals? ); R^2^ = 0.98). No significant differences were detected in the overall linear increase of seizures/week after rTMS compared to pre rTMS in non-improving dogs (ANCOVA, F(1, 22) = 0.21, *p* = 0.65) indicating no aggravation of symptoms following rTMS.

**Table 3:** Comparison of results between the 5-day and 3-day repetitive transcranial magnetic stimulation protocol post-rTMS

	5-day protocol (*n* = 12)		3-day protocol (*n* = 15)	
	MSF	MSDF	MSF	MSDF
Overall change in seizure parameters	− 44%	− 42%	− 19%	− 22%
Number of animals with decrease in seizure parameters	12/12 (100%)	12/12 (100%)	7/15 (47%)	8/15 (53%)
Number of responders (50–100% reduction)	5/12 (42%)	6/12 (50%)	4/15 (27%)	4/15 (27%)

## Discussion

Two former studies showed that a 3-day or a 5-day rTMS is an effective treatment for DRE [18, 20]. The aim of the current analysis was to perform an exploratory analysis and critically review the degree of treatment responsiveness between two published rTMS studies. While the studies differ slightly in design, their stimulation parameters are largely comparable, allowing for a meaningful evaluation of protocol-specific effects. The primary distinction between the protocols lies in the distribution of the total 8100 pulses, with 2700 pulses administered daily in the 3-day protocol and 1620 pulses administered daily in the 5-day protocol. While both protocols were safe and effective, the 5-day protocol yielded a significantly better reduction in seizure activity, with a 44% decrease, compared to the 3-day protocol resulting in a 19% reduction. Notably, all animals in the 5-day group exhibited a decrease in seizure frequency, whereas some animals in the 3-day group demonstrated an increase or no change in seizure frequency. In addition, the number of responders (seizure activity reduction of ≥ 50%) was significantly higher in the 5-day protocol compared to the 3-day protocol.

To rule out a possible increase in seizure susceptibility due to rTMS, regression analyses among non-responders have shown that the cumulative increase in seizure parameters was consistent both before and after stimulation. Therefore, it could be concluded that rTMS did not lead to a potential acceleration of disease progression in these animals, providing a clear indication of the safety of the method. Seizure frequency can vary overtime [23], also in DRE patients. Especially in patients recruited for trials testing new management concepts for DRE, animals with progressively higher seizure frequencies are being included, which leads to a selection bias [24]. The cumulative seizure data (Fig. 3) indicated that rTMS significantly reduced seizure occurrence in animals with improvement and did not aggravate seizure frequency in the dogs not having any improvement in seizure control. This is supported by a study in humans with and without epilepsy, which confirmed rTMS safety [25].

To the best of our knowledge, no available studies have conclusively determined whether extending the treatment duration beyond a single day leads to a significant difference in the effectiveness of rTMS in the treatment of epilepsy. Evidence from studies in other disorders, such as depression in humans, suggests that multi-day treatment protocols may produce greater effects than longer single-day stimulation sessions [26]. In epilepsy, various controlled clinical studies have employed differing treatment regimens, with the majority conducting rTMS over five consecutive days; however, protocols involving a total of ten stimulation days distributed across two weeks have also been reported [27–32]. Nonetheless, direct comparison is limited due to varying stimulation parameters, including intensity, coil type, focal versus non-focal stimulation, and the total number of pulses administered [27, 28, 31, 33]. Previous investigations into the duration of stimulation have predominantly focused on these factors, with limited attention given to the impact of distributing pulses over varying numbers of treatment days. To date, no study in veterinary medicine has specifically addressed whether the temporal distribution of pulses influences the efficacy of rTMS.

In human medicine, it is well-established that various factors can influence the outcome of rTMS, including the accessibility of the epileptic focus, with superficial structures being more amenable to treatment than deep structures. This is supported by studies demonstrating improved outcomes in patients with neocortical foci compared to those with mesial temporal foci [28–30, 32]. Additionally, it is crucial whether the epileptic focus is focally stimulated or whether a non-focal stimulation is performed. There are indications that focal stimulation could lead to a stronger reduction in seizure frequency [27]. In the studies presented here, the epileptic focus was not determined and a non-focal stimulation using a circular coil was therefore performed.

The shape of the coil could also have an impact on the outcome of rTMS, although a study from 2016 showed that there is no significant difference in the application of an 8-shaped coil versus a circular coil [31]. In the current study, two circular coils were used, which differed in diameter (12 cm vs. 15 cm) [18, 20]. It was formerly suggested that a longer daily stimulation period (3000 pulses/day vs. 1500 pulses/day) could improve the outcome of the seizure suppressing effect [27]. This was not confirmed in the current study. The intensity of the coil output should also play a crucial role in the outcome, with a higher output showing a significant improvement in seizure control compared to a lower output [28]. In the current study, the coil output was individually adjusted to the patient corresponding to 200% of the individually determined motor cortex threshold [18, 20]. However, a larger temporal extension of the stimulation across various days appeared to have a greater importance for seizure control than the coil output or the number of pulses per day.

Various studies from human medicine show no consensus on the effectiveness of rTMS, with some highlighting a significant reduction in seizure frequency, whereas others do not observe such a reduction [27–33]. The Cochrane library assessment states that the evidence remains low in people with DRE for the primary outcome of rTMS, being reduction in seizure frequency [17]. In a 2015 study, participants who received rTMS did not exhibit a significant reduction in seizure frequency, but they reported a significant improvement in quality of life, suggesting that rTMS may have additional benefits beyond seizure control [31]. In other studies, a significant reduction in epileptiform discharges was reported [27–29]. The dogs in this study are refractory patients that have received two or more ASMs in optimal dosages and/or with optimal serum concentrations yet continue to exhibit seizure activity. Despite the therapeutic difficulty with DRE patients [34] rTMS can provide opportunities to circumvent possible therapeutic boundaries.

The rationale for using a 3-day protocol was to improve the acceptability of rTMS as a treatment for DRE among owners. In contrast to human medicine, veterinary patients must be sedated during rTMS treatment, as they need to remain still throughout the procedure. This makes rTMS treatment time- and cost-intensive, posing challenges for owners and veterinary practices. Furthermore, the procedure may cause stress to the patients, who may need to be hospitalised for the duration of the treatment or have a new intravenous access inserted daily to administer sedation. For practical reasons, a shorter protocol would be desirable, as it would reduce stress on the animals, staffing, and other drugs’ and consumables’ costs associated with treatment. Although the longer 5-day protocol may offer potentially better efficacy in patients, the advantage of the 3-day protocol lies in its improved feasibility. The fact that the 3-day protocol is effective as well as shorter in duration can make it appealing as a treatment option. Additionally, some patients were able to achieve seizure freedom. Therefore, the choice of the appropriate patient protocol should be made individually, considering both the patient’s needs and practical considerations.

An important limitation of this exploratory study is that, although the two underlying studies shared a largely comparable design and similar stimulation parameters, there were differences regarding the research sites, time points, populations, and brands of equipment. These differences may have influenced the focality and intensity of the magnetic field, potentially impacting the stimulation effects and treatment outcomes. Regarding the equipment, differences in the manufacturers and coils might play a role to the rTMS effect. However, both studies used the same type of coils (circular), which are well-suited for generalized epileptic seizures and stimulate a larger portion of the cortex [16, 27, 33, 35, 36]. Furthermore, this analysis is retrospective and relies on owner-reported seizure data, which may be incomplete or imprecise, as owners are not always able to observe or accurately record seizures continuously. Electroencephalography (EEG) represents the gold standard for seizure detection, minimizing the risk of unrecognized seizure activity; however, in veterinary medicine, no standardized electrode placement comparable to that used in human medicine has yet been established [37, 38] .The follow-up period in this study was limited to three months, which represents a relatively short duration and may not fully capture the long-term efficacy and sustainability of seizure control achieved by rTMS treatment. To advance the understanding of optimal stimulation parameters for canine epilepsy, larger-scale prospective studies are warranted. Future controlled trials should aim to consider additional biological and environmental variables that may influence seizure susceptibility and treatment response. Factors such as neurodevelopmental stage, sex-related and hormonal influences, including seizure modulation by the ovarian cycle, as well as circadian rhythms and time of treatment administration, could play a significant role in therapeutic outcomes. Previous studies have demonstrated circadian and multiday seizure periodicities in dogs with idiopathic epilepsy [39] and a possible association between estrus and seizure occurrence in intact bitches [40] underscoring the relevance of these parameters for future rTMS investigations. Furthermore, the use of electroencephalography could facilitate the identification of the epileptic focus in the brain, enabling a more targeted selection of patients and stimulation of the affected brain region, thereby maximising treatment success. Another limitation was the combination of data from blinded, randomised, sham-controlled trials and non-randomised, uncontrolled trials. This may introduced a risk of bias.

## Conclusion

In conclusion, the rTMS stimulation protocols compared in this exploratory study resulted in a significant reduction in seizure frequency in both the 5-day and 3-day groups. Notably, the 5-day protocol appeared to yield better outcomes in terms of MSF and MSDF in dogs with drug-resistant idiopathic epilepsy. Furthermore, rTMS was shown not to aggravate epilepsy progression in animals with no treatment improvement, supporting the safety of the method. Since the 3-day protocol is effective as well as shorter in duration, it might be a more feasible treatment option, compared to the 5-day protocol, for the owners and their dogs. However, due to the small sample size and varying baseline seizure frequencies, larger-scale prospective clinical studies are necessary to confirm these findings and to establish the optimal treatment duration. Furthermore, future research is required to elucidate the factors that contribute to individual variability in response to rTMS treatment, including the role of seizure focus accessibility and other potential predictors of treatment outcome.

## Data Availability

The datasets used and/or analysed during the current study are available from the corresponding author on reasonable request.

## Abbreviations

rTMS: Repetitive transcranial magnetic stimulation
MSF: Monthly seizure frequency
MSDF: Monthly seizure day frequency
ASMs: Antiseizure medications
GTCS: Generalised tonic-clonic seizures
PB: Phenobarbital
KBr: Potassium bromide
Leve: Levetiracetam
Imp: Imepitoin
Hz: Hertz
